# Vestibular rehabilitation for persons with stroke and concomitant dizziness—a pilot study

**DOI:** 10.1186/s40814-020-00690-2

**Published:** 2020-09-30

**Authors:** Eva Ekvall Hansson, Hélène Pessah-Rasmussen, Annika Bring, Birgit Vahlberg, Liselott Persson

**Affiliations:** 1 Department of Health Sciences, Division of Physiotherapy, Lund University, Health Science Centre, Box 157, 221 00 Lund, Sweden; 2 Department of Neurology and Rehabilitation Medicine, Skåne University Hospital, Lund, Sweden; 3 Department of Clinical Sciences, Lund University, Lund, Sweden; 4 Academic Primary Health Center, Primary Care and Health, Uppsala County Council, Uppsala, Sweden; 5 Department of Neuroscience, Physiotherapy, Uppsala University, Uppsala, Sweden; 6 Department of Public Health and Caring Sciences, Clinical Nutrition and Metabolism, Uppsala University, Uppsala, Sweden

## Abstract

**Background:**

Dizziness is common among patients with first time stroke. It affects self-perceived health and is a risk factor for falls. Vestibular rehabilitation (VR) is effective for treating dizziness among various conditions, but the effect of dizziness with origin in the central nervous system is poorly studied.

This pilot study of a randomized controlled trial aimed at investigating a vestibular rehabilitation programme among patients with first time stroke and concomitant dizziness. A second aim was to study the feasibility of performing the randomized controlled trial.

**Methods:**

The participants were computer generated randomized to either an intervention or a control group. The intervention comprised of four different vestibular rehabilitation exercises, adapted for each patient and usual rehabilitation. The control group received usual rehabilitation without the vestibular rehabilitation exercises. Outcome measures used were The Activities-specific Balance Confidence Scale, the Berg Balance Scale, the Functional Gait Assessment Scale and the EuroQol-5D. Feasibility was studied in terms of recruitment, adherence and retention rates, also as the ability to collect primary and secondary outcomes as well as to find indications of treatment differences.

**Results:**

Self-rated health improved for all participants. No other differences between baseline and follow-up were detected neither within nor between groups. Recruitment rate was 23%, adherence to the intervention 90%, retention rate 69% and ability to collect outcome measures 90%. No adverse events occurred.

**Conclusion:**

Both the intervention and the control groups improved in self-perceived health. The measures of feasibility were satisfactory in this study, apart from a low recruitment rate.

## Introduction

Vertigo and dizziness are the cognitive manifestation of a disturbance in the balance system [1]. Commonly, vertigo can be described as an illusion of movement or as if the body or surroundings are spinning like a carousel [2]; dizziness is used as a non-specific term for describing sensations such as vertigo, presyncope, disequilibrium and lightheadedness. Hence, balance and dizziness are closely related and balance measures are included in the assessment of dizzy patients [3].

There is a relation between dizziness and stroke; dizziness as the only symptom preceding a stroke is unusual [4], but patients who are hospitalized for vertigo are at higher risk for stroke than the general population [5]. Also, patients with dizziness or vertigo who have been discharged from an emergency unit are at an increased risk of subsequent vascular events [6]. Recently, our research group has shown that as many as 70% of patients with first time stroke had dizziness and that the risk of being dizzy after a stroke is higher among women than men. We also showed that dizziness negatively affects self-perceived health [7]. Furthermore, dizziness is a risk factor for falls [8] and has shown to have a negative influence on the quality of life [9].

Vestibular rehabilitation programmes were first developed in the 1940s and used to facilitate recovery from peripheral vestibular disorders [10, 11]. Modern research has widened the use of vestibular rehabilitation to patients with other causes of dizziness than peripheral vestibular disorders [12–15], and there are now indications that vestibular rehabilitation can also have a positive effect on dizziness and vertigo with origin from the central nervous system, such as stroke [16, 17]. Vestibular rehabilitation has also shown to have positive effects on gait among persons with stroke [18]. However, studies concerning the effect of vestibular rehabilitation on dizziness, balance and self-perceived health among persons who have suffered a stroke seem to be lacking. However, before conducting a full-scale randomized controlled trial, pilot-testing is needed.

The aim of this study was initially to perform a full RCT, but since the recruitment rate was very slow, the decision to change the aim to a pilot study was made. Therefore, the aim was to investigate a vestibular rehabilitation programme among patients with first time stroke and concomitant dizziness and also to study the feasibility of performing the randomized controlled trial in terms of recruitment, compliance and retention rates, and the ability to collect primary and secondary outcomes as well as to find indications of treatment differences.

## Method

The study was initially designed as a full RCT, registered in ClinicalTrials.gov, number NCT01797744. Since recruitment rate was very slow, the decision to change the aim to a pilot study was made.

### Participants and setting

Inclusion criteria for participating in the project were the same as in an earlier study [6]: first ever stroke survivors with concomitant dizziness and/or unsteadiness, 18 years or older who have attended physiotherapy or occupational therapy for post-stroke rehabilitation and who are physically able to participate in a vestibular rehabilitation programme and also have an adequate ability to understand and speak the Swedish language. The exclusion criterion was having other disorders that could affect dizziness, such as Mb Ménière or vestibular schwannoma. Dizziness was defined by a question about the type of dizziness (rotational or spinning, unsteadiness, others or do not know). These questions have been used in other studies on dizziness incidence [7, 19]. To establish if dizziness was present before or after the stroke, a question addressing this was also asked.

Participants in the study were recruited from primary health care, from a geriatric clinic and from neurological rehabilitation centres. Recruitment to the study commenced in March 2013 and was terminated in December 2017.

### Outcomes

#### Primary outcome measure

The Activities-specific Balance Confidence Scale (ABC-scale) was used as the primary outcome measure for balance [20]. The ABC-scale consists of 16 activities with varying levels of difficulty, and the responder rates how confident he or she feels when performing these activities on a scale from 0 to 100%, lower levels indicating lower level of confidence. The scale has shown to have moderate significant positive correlation with the Berg Balance Scale (BBS) and to have a good internal consistency for patients with stroke [21]. The ABC-scale has been translated to Swedish, and the Swedish version has shown to have good test–retest reliability [22]. The ABC-scale has proven to be able to identify the influence that vestibular dysfunction can have on daily activities [23].

#### Secondary outcome measures

The Berg Balance Scale (BBS) [24] and the Functional Gait Assessment (FGA) [25] were used in addition to the ABC-scale for measuring balance. The BBS consists of 14 different tasks related to balance. The 14 tasks are graded from 0 (unable to perform) to 3 (normal). BBS has shown to have good psychometric qualities to assess rehabilitation for patients with stroke [21]. FGA consists of 10 items assessing balance during walking, and each item is graded from 0 (severe impairment) to 3 (normal). FGA has shown excellent inter-rater, intra-rater and test–retest reliability when used on patients with stroke [26].

To measure function and self-rated health, the generic instrument EuroQol-5D (EQ5D) was used [27]. EQ5D consists of a descriptive system comprising five dimensions: mobility, self-care, usual activities, pain/discomfort and anxiety/depression. Each dimension has three levels: no problems, some problems and severe problems. The patient indicates his/her state by marking the most appropriate statement. This returns a one-digit number, and the digits for the five dimensions can be combined in a five-digit number, generating 243 possible response combinations. The EQ5D can be presented as a health profile or as a global health index with a weighted total value (European tariff used), with the minimum value being − 0.594 and the maximum 1.0 [28]. EQ5D has been tested for validity when measuring the quality of life after a stroke [29]. In the EQ VAS, the patients record self-rated health on a vertical, visual analogue scale where the individual points can be used as a quantitative measure with 0 as worst rated health and 100 as best rated health [27].

#### Feasibility outcomes

*Recruitment rate* was calculated as the percentage of participants that accepted to participate in the study among those who were estimated to be eligible. The estimation was calculated by using data from two previous studies [7, 30]. *Compliance* was calculated as the percentage of the total number of training sessions completed by each participant in each arm. *Retention rate* was calculated as the percentage of enrolled participants that completed the primary follow-up assessments. Reasons for non-completion of allocated treatment or primary follow-up were also collected. The *ability to collect outcome assessment* was calculated as the percentage of possible outcome measures completed at baseline and follow-up. *Adverse events* were operationalized as the number of harmful, negative or unfavourable events that may have influenced the study procedure in any way.

### Procedure

Patients who were attending a regular visit for post-stroke rehabilitation with a physiotherapist (PT) or occupational therapist (OT) in primary health care or neurological rehabilitation centres in the region of Skane or at the Geriatric day-care department, Uppsala University Hospital, Sweden, were invited to participate in the study. Those patients who accepted to participate were assessed by an independent PT. The participants were then computer generated randomized to either an intervention or the control group. The coded randomization results were placed in sealed envelopes. Rehabilitation for both the intervention group and the control group was performed by other independent PTs who did not perform the baseline or follow-up assessments. All patients were planned to train twice a week for 3 months. After 3 months, all patients were assessed again, by the same blinded PT as at baseline. Adherence to the intervention was noted by the PT who performed the intervention and then distributed to the primary investigator (EEH).

### Intervention

The intervention was an add-on to usual rehabilitation and comprised four different vestibular rehabilitation exercises, adapted for each patient (Table 1). Feasibility of the intervention was assessed in terms of effects in smaller samples [31].

**Table 1:** The four vestibular exercised included in the intervention group, a–e indicating progression in difficulty of the exercise

Standing on a padded mat, feet as close to each other as the patient was able to. a. Move your eyes from right to left b. Close your eyes c. Close your eyes and move your head from right to left	
Sitting on a Swiss ball. a. Feet on the floor, bounce up and down on the ball, move your eyes to the right and left b. Feet on the floor, bounce up and down on the ball, move your head from right to left c. As above with closed eyes d. As above (a + b + c) but with your feet on a wobble board	
Standing on a trampoline. a. Feet wide apart and bounce up and down b. Legs wide apart and bounce up and down while moving your eyes from right to left c. As above, with closed eyes d. As above, but also move your head up and down e. As above, but with your feet close to each other	
Sitting on a chair or on the edge of a bed. a. Stand up and sit down, looking from right to left at the same time b. As above, with head movements to the right and left c. As above, with head movements to the right and left, and with eyes closed d. As above, (a + b + c) with feet on a padded mat	

The control group performed usual rehabilitation without the additional vestibular rehabilitation exercises.

For both groups, usual rehabilitation comprised of individually adapted exercises, based on the assessment by the treating physiotherapist.

### Statistical analysis

The a priori power calculation revealed that, for a power of 80% at significance level 0.05, a sample size between 30 and 40 persons in each group was deemed to be required (30 for ABC-scale and 40 for BBS) [32] for a RCT. Since this study was changed from a full RCT to a pilot study, we used available patients, which makes a sample size of 30% of the full-size RCT. Considering the small sample size, the Mann-Whitney U test was used to test for differences between the two groups and within groups for quantitative data and chi-square or Fisher’s test when calculating for differences in proportions. Ninety-five per cent CI was calculated. To follow the CONSORT statement, an intention-to-treat analysis was applied.

The Data package SPSS version 23.0 was used (SPSS Inc., software location Lund University).

### Ethics

All patients who agreed to participate in the study gave their informed consent. The study was approved by the regional ethics committee in Lund (number 2012/816).

## Results

A total of 32 persons were included in the study, 19 women and 13 men aged 32 to 85 years (mean 68). Mean time since stroke was 8.5 months (SD 10.9), with a total range between 1 and 60 months. Sixteen persons had a right-sided paresis, 11 a left-sided and 5 persons had no paresis. Two persons had experienced dizziness already before stroke while 30 had become dizzy after stroke. The most common type of dizziness was unsteadiness (*n* = 25), while four persons had rotational/spinning type of dizziness and three persons had a combination of unsteadiness and rotational/spinning type.

**Fig. 1:**
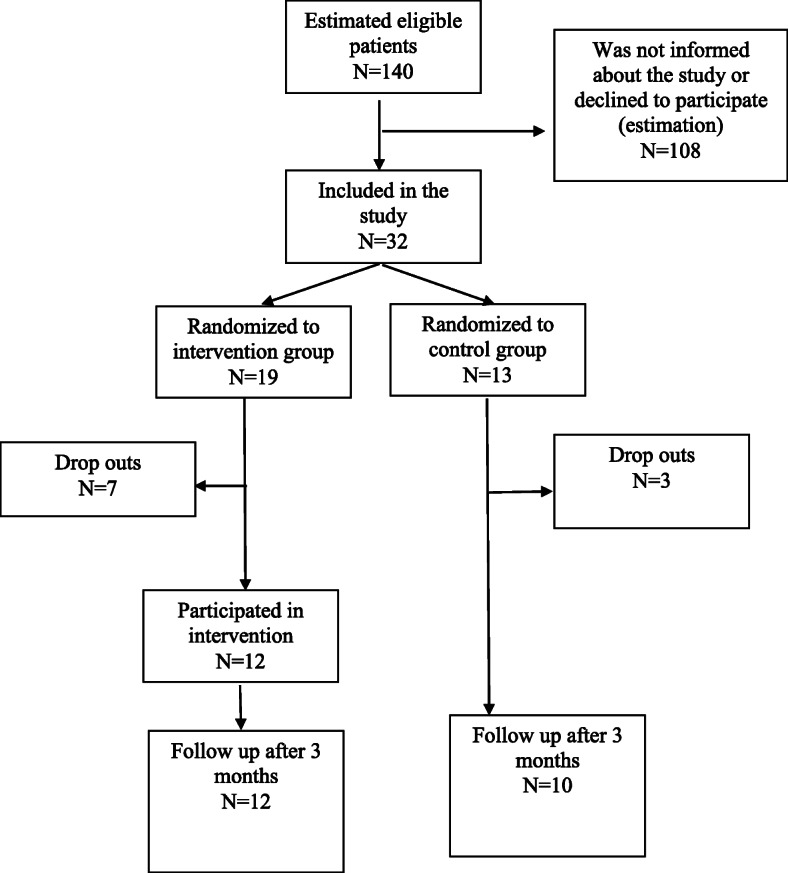
Flow chart of the study

Among the 32 persons included in the study, nineteen persons were randomized to the intervention group and 13 to the control group. A flow chart of the study is shown in Fig. 1, and baseline measures for the total group as well as divided into intervention and control groups are given in Table 2.

**Table 2:** Median and interquartile range (IQR) for baseline measures for the total group and divided into intervention and control groups

Measure	Total sample ***n*** = 32	Intervention group ***n*** = 19	Control group ***n*** = 13
Women/men	19/13	11/8	8/5
Age (years)	68 (10.9)	71 (15)	69 (14)
ABC	59.0 (41.82)	59.3 (43.12)	55 (42.57)
BBS	42.5 (14.0)	44 (12)	41 (17.5)
FGA	20 (13)	20 (11)	21.5 (18.75)
EQ5D index	0.59 (0.17)	0.58 (0.17)	0.59 (0.18)
EQ5DVAS in mm	57 (30)	69 (31)	50 (28.5)

*ABC* Activities-specific Balance Confidence Scale, *BBS* Berg Balance Scale, *FGA* Functional Gait Assessment, *EQ5D index* EuroQol 5 Dimension index, *EQ5D VAS* EuroQol 5 Dimension Visual Analogue Scale

### Differences between baseline and follow-up

#### Within-group differences

Both groups improved in self-rated health, measured with EQ5D and VAS (*p* < 0.00 and *p* = 0.04 respectively). There were no significant differences in any of the other measures between baseline and follow-up in any of the groups (Table 3).

**Table 3:** Intention-to-treat analysis of differences in change between baseline and follow-up between and within groups

	Between-group differences, intervention (***n*** = 19) versus control (***n*** = 13)			Within-group differences in the intervention group ***n*** = 19			Within-group differences in the control group ***n*** = 13		
Measure	Median change	(CI) for median change	***p*** value for median change*	Median change	(CI) for median change	***p*** value for median change	Median change	(CI) for median change	***p*** value for median change
ABC	0.00	(0.00–8.44)	0.705	0.00	(− 0.55–2.80)	0.279	0.00	(0.00–16.07)	0.484
BBS	0.00	(− 4.00–0.00)	0.734	0.00	(0.00–3.00)	0.361	0.00	(− 2.00–6.00)	0.154
FGA	0.00	(0.00–1.00)	0.484	0.00	(0.00–0.00)	0.683	0.00	(0.00–2.00)	0.345
EQ5D index	0.07	(0.06–0.16)	0.483	0.08	(0.06–0.20)	**0.001**	0.06	(0.04–0.14)	**0.005**
EQ5D VAS	5.00	(0.00–20.00)	0.850	5.00	(0.00–15.00)	**0.003**	2.00	(0.00–21.00)	**0.036**

*ABC* Activities-specific Balance Confidence Scale, *BBS* Berg Balance Scale, *FGA* Functional Gait Assessment, *EQ5D index* EuroQol 5 Dimension index, *EQ5D VAS* EuroQol 5 Dimension Visual Analogue Scale

*p values calculated by the Mann-Whitney U test

#### Between-group differences

There were no differences between the two groups regarding changes in values between baseline to 3-month follow-up in any of the measures (*p* = 0.51–0.70) (Table 3).

### Feasibility measures

#### Recruitment

Based on our calculation that it was likely that about 200 persons with stroke [30] would see a PT in any of the centres involved in the study and that 70% of these also would have had dizziness [7], the recruitment percentage was 23%. Efforts were made to increase the number of participants during the 5-year long period, such as several information meetings at the different clinics where patients could be recruited, reminding e-mails to contact persons at the clinics and personal communication with the contact persons. Despite this, the recruitment rate was slow and the a priori sample size for a full RCT was not achieved.

#### Compliance

The individuals’ (*n* = 12) compliance to the intervention ranged between 50 and 100% with a median value of 90%.

#### Retention rates

Twenty-two out of thirty-two participants (69%) completed the primary follow-up assessments. Ten participants dropped out of the study. Reasons for dropout were unknown reason (*n* = 4), other sickness (*n* = 3), disappointment over being randomized to the control group (*n* = 2) and referred to rehabilitation elsewhere (*n* = 1).

#### Collecting primary and secondary outcomes

At baseline, 99% of the possible outcome measures was collected (ABC 100%, BBS 100%, EQ5D index 97% and EQ5DVAS 100%) (*n* = 32).

At follow-up, 66% of the possible outcome measures was collected (ABC 69%, BBS 69%, EQ5D index 60% and EQ5DVAS 66%) (*n* = 22).

#### Adverse events

One adverse event was observed that may have negatively influenced the study procedure: namely that two participants dropped out because of disappointment over being randomized to the control group.

No actual harmful event occurred during the study period.

## Discussion

This pilot study showed that both the intervention group and the control group improved self-rated health, measured by EQ-5D. No other significant changes were found, neither differences between the groups. The study also demonstrated that compliance to the intervention, retention rate and ability to collect data were high. No adverse events that were considered harmful for the participants occurred, but the recruitment rate was low.

Our findings confirm that results regarding VR for persons with stroke and concomitant dizziness and vertigo are conflicting. There are studies showing improvements in gait performance [18, 33] and others showing no difference in balance and gait performance between VR and no VR groups [34]. Small sample size might be one explanation for the lack of difference between the groups in our study. Other possible explanations might be the large variance in time since stroke onset, the choice of our outcome measures or the fact that participating in the study, regardless of what group, could have empowered the participants in relation to how they experience their dizziness.

Recruiting participants in rehabilitation RCTs can be problematic, and regular engagement with the entire stroke multidisciplinary team is recommended [35]. In the present study, much effort was put on regular meetings with the PTs engaged in the study; however, we did not have the opportunity to meet the total multidisciplinary team, which can be one reason for the low recruitment rate. Another reason could be that many physiotherapists had knowledge of vestibular rehabilitation as treatment for vertigo and dizziness and used the method already. Thus, they were less willing to recruit participants to the study, risking their patient to be randomized to the control group and not get the intervention that the physiotherapist already considered the patient in need of, even if evidence for the method is missing. Other reasons for the low recruitment rate could be difficulties in finding clinical routines that enhanced recruitment or that patients were informed about the study but chose not to participate; however, this has not been investigated in this study.

A similar small study with some different methodology, interventions and primary outcome demonstrated that individuals with neglect after right hemispheric stroke, through participation in VR, improved regarding the degree of neglect, ADL and balance [36]. However, both the VR and control groups in that study improved in balance but there were no statistically significant differences in improvement between the groups and they did not use data on self-rated health. No effect on fall incidence was seen.

On the other hand, another small study with 25 participants found VR for individuals with subacute stroke to be effective for improving gait speed, stride length and dynamic balance compared to a control group [18]. Improvements were obtained from participating in a 4-week VR programme consisting of balance exercises for the controls and gaze stability and postural exercises for individuals in the VR group.

Acute vestibular syndrome due to stroke is mostly common with lesions in the cerebellum and in the brainstem [37, 38]. Individuals with stroke and cerebellar symptoms such as ataxia, unsteadiness and vertigo might most likely benefit from exercises directed at the vertigo symptoms. Brainstem lesions often present various vestibular signs and nystagmus as important findings [37, 38]. Visual impairment, sensory impairments and muscle weakness can also cause a feeling of unsteadiness and vertigo. The possible multifactorial cause of dizziness among persons with stroke is also the reason why the ABC-scale was used as the primary outcome measure. Other scales measuring dizziness/vertigo are available, such as the Dizziness Handicap Scale [39] or the Vertigo Symptom Scale [40]. However, it might be difficult to distinguish whether the symptom relates to dizziness/vertigo or to other stroke-related dysfunction. The ABC-scale can detect how vestibular dysfunction influences daily activities, and was therefore considered as an appropriate measure in this study.

Due to the possible multifactorial cause of dizziness/vertigo among persons with stroke, randomized controlled trials involving larger samples are required and also studying the intervention in the acute, subacute and chronic phase post-stroke. It can also be speculated that the VR training should start as soon as the acute stroke patient is medically stable, which was not the case in the present study.

The current study has limitations that need to be addressed. Individuals were included without considerations to brain ischaemic lesion sites, which might have an impact on the results. In addition, the original design of the study was a full RCT, not a pilot study also including measures of feasibility. Thus, we had not the opportunity to collect all feasibility measures, for example how the patients in the intervention group perceived the intervention. The strengths of this study are its randomized controlled design and exercise protocol which may facilitate the design and planning of a future larger RCT.

In conclusion, both the intervention and the control groups improved in self-perceived health and measures of feasibility were satisfactory in this study, apart from the low recruitment rate. Based on these results, a larger multicentre RCT study may be suggested with an even more thorough preparation concerning recruitment logistics at each site and that efforts to engage the total stroke multidisciplinary team to recruit participants are made.

## Data Availability

The datasets during and/or analysed during the current study are available from the corresponding author on reasonable request.

## References

[1] Magnusson M, Strupp M (2010). A short guide to practical management of the dizzy patient: Solvay.

[2] Alyono JC (2018). Vertigo and dizziness: understanding and managing fall risk. Otolaryngol Clin N Am.

[3] Hansson EE, Månsson NO, Håkansson A (2005). Balance performance and self-perceived handicap among dizzy patients in primary health care. Scand J Prim Health Care.

[4] Kerber KA, Meurer WJ, West BT, Fendrick AM (2008). Dizziness presentations in U.S. emergency departments, 1995-2004. Acad Emerg Med.

[5] Lee CC, Su YC, Ho HC, Hung SK, Lee MS, Chou P, et al. Risk of stroke in patients hospitalized for isolated vertigo. A four-year follow-up study. Stroke. 2010. 10.1161/STROKEAHA.110.597070 21127296

[6] Lee CC, Ho HC, Su YC, Chiu BC, Lee YD, Chou P (2012). Increased risk of vascular events in emergency room patients discharged home with diagnosis of dizziness or vertigo: a 3-year follow-up study. PLoS One.

[7] Hansson EE, Beckman A, Naslund A, Persson S, Janson S, Troein M (2014). Stroke and unsteadiness - a cross-sectional study from primary health care. NeuroRehabilitation..

[8] Moreland J, Richardson J, Chan DH, O'Neill J, Bellissimo A, Grum RM (2003). Evidence-based guidelines for the secondary prevention of falls in older adults. Gerontology..

[9] Grimby A, Rosenhall U (1995). Health-related quality of life and dizziness in old age. Gerontology..

[10] Cawthorne T (1945). The physiological basis for head exercises. J Chartered Soc Physiother.

[11] Cooksey I (1946). Rehabilitation in vestibular injuries. Proc R Soc Med.

[12] Hansson EE (2007). Vestibular rehabilitation - for whom and how?. Adv Physiother.

[13] Hansson EE, Mansson NO, Hakansson A (2004). Effects of specific rehabilitation for dizziness among patients in primary health care. A randomized controlled trial. Clin Rehabil.

[14] Hansson EE, Månsson N-O, Ringsberg K, Håkansson A (2006). Dizziness among patients with whiplash associated disorder - a randomized controlled trial. J Rehabil Med.

[15] Kammerlind A, Håkansson J, Skogsberg M (2001). Effects of balance training in elderly people with nonperipheral vertigo and unsteadiness. Clin Rehabil.

[16] Brown KE, Whitney SL, Marchetti GF, Wrisley DM, Furman JM (2006). Physical therapy for central vestibular dysfunction. Arch Phys Med Rehabil.

[17] Cowand JL, Wrisley DM, Walker M, Strasnick B, Jacobson JT (1998). Efficacy of vestibular rehabilitation. Otolaryngol Head Neck Surg.

[18] Tramontano M, Bergamini E, Iosa M, Belluscio V, Vannozzi G, Morone G (2018). Vestibular rehabilitation training in patients with subacute stroke: a preliminary randomized controlled trial. NeuroRehabilitation..

[19] Gopinath B, McMahon CM, Rochtchina E, Mitchell P (2009). Dizziness and vertigo in an older population: the Blue Mountains prospective cross-sectional study. Clin Otolaryngol.

[20] Botner EM, Miller WC, Eng JJ (2005). Measurement properties of the Activities-specific Balance Confidence Scale among individuals with stroke. Disabil Rehabil.

[21] Blum L, Korner-Bitensky N (2008). Usefulness of the Berg Balance Scale in stroke rehabilitation: a systematic review. Phys Ther.

[22] Jarlsäter S, Mattsson E (2003). Test of reliability of the Dizziness Handicap Inventory and The Activities-specific Balance Confidence scale for use in Sweden. Adv Physiother.

[23] Whitney SL, Hudak MT, Marchetti GF (1999). The activities-specific balance confidence scale and the dizziness handicap inventory: a comparison. J Vestib Res.

[24] Berg K, Maki B, Williams J, Maki B (1992). Measuring balance in the elderly: validation of an instrument. Can J Public Health.

[25] Wrisley DM, Marchetti GF, Kuharsky DK, Whitney SL (2004). Reliability, internal consistency, and validity of data obtained with the functional gait assessment. Phys Ther.

[26] Van Bloemendaal M, Bout W, Bus SA, Nollet F, Geurts AC, Beelen A. Validity and reproducibility of the Functional Gait Assessment in persons after stroke. Clin Rehabil. 2018;269215518791000. 10.1177/0269215518791000 30084264

[27] EQ5D. [Available from: http://www.euroqol.org/.

[28] Dolan P (1997). Modeling valuations for EuroQol health states. Med Care.

[29] Dorman PJ, Waddell F, Slattery J, Dennis M, Sandercock P (1997). Is the EuroQol a valid measure of health-related quality of life after stroke?. Stroke.

[30] Hansson EE, Beckman A, Wihlborg A, Persson S, Troein M. Rehabilitation after stroke - a retrospective study of satisfaction with rehabilitation in relation to health and function among patients with stroke. Scand J Caring Sci. 2012; In press. 10.1111/j.1471-6712.2012.01041.x 22804807

[31] Thabane L, Ma J, Chu R, Cheng J, Ismaila A, Rios LP (2010). A tutorial on pilot studies: the what, why and how. BMC Med Res Methodol.

[32] Altman D (1991). Practical statistics for medical research.

[33] Mitsutake T, Sakamoto M, Ueta K, Oka S, Horikawa E (2017). Effects of vestibular rehabilitation on gait performance in poststroke patients: a pilot randomized controlled trial. Int J Rehabil Res.

[34] Balci BD, Akdal G, Yaka E, Angin S (2013). Vestibular rehabilitation in acute central vestibulopathy: a randomized controlled trial. J Vestib Res.

[35] Tyson SF, Thomas N, Vail A, Tyrrell P (2015). Recruiting to inpatient-based rehabilitation trials: lessons learned. Trials..

[36] Dai CY, Huang YH, Chou LW, Wu SC, Wang RY, Lin LC (2013). Effects of primary caregiver participation in vestibular rehabilitation for unilateral neglect patients with right hemispheric stroke: a randomized controlled trial. Neuropsychiatr Dis Treat.

[37] Choi KD, Lee H, Kim JS (2013). Vertigo in brainstem and cerebellar strokes. Curr Opin Neurol.

[38] Lee H, Sohn SI, Cho YW, Lee SR, Ahn BH, Park BR (2006). Cerebellar infarction presenting isolated vertigo: frequency and vascular topographical patterns. Neurology..

[39] Jacobson GP, Newman CW (1990). The development of the Dizziness Handicap Inventory. Arch Otolaryngol Head Neck Surg.

[40] Yardley L, Masson E, Verschuur C, Haacke N, Luxon L (1992). Symptoms, anxiety and handicap in dizzy patients: development of the vertigo symptom scale. J Psychosom Res.

